# Increasing RpoS Expression Causes Cell Death in *Borrelia burgdorferi*

**DOI:** 10.1371/journal.pone.0083276

**Published:** 2013-12-16

**Authors:** Linxu Chen, Qilong Xu, Jiagang Tu, Yihe Ge, Jun Liu, Fang Ting Liang

**Affiliations:** 1 Department of Pathobiological Sciences, Louisiana State University, Baton Rouge, Louisiana, United States of America; 2 Department of Pathology and Laboratory Medicine, University of Texas Medical School at Houston, Houston, Taxes, United States of America; Cornell University, United States of America

## Abstract

RpoS, one of the two alternative *σ* factors in *Borrelia burgdorferi*, is tightly controlled by multiple regulators and, in turn, determines expression of many critical virulence factors. Here we show that increasing RpoS expression causes cell death. The immediate effect of increasing RpoS expression was to promote bacterial division and as a consequence result in a rapid increase in cell number before causing bacterial death. No DNA fragmentation or degradation was observed during this induced cell death. Cryo-electron microscopy showed induced cells first formed blebs, which were eventually released from dying cells. Apparently blebbing initiated cell disintegration leading to cell death. These findings led us to hypothesize that increasing RpoS expression triggers intracellular programs and/or pathways that cause spirochete death. The potential biological significance of induced cell death may help *B. burgdorferi* regulate its population to maintain its life cycle in nature.

## Introduction

The Lyme disease spirochete *Borrelia burgdorferi* maintains its life cycle by traveling between the tick vector and a mammal. After acquired by a larval tick, *B. burgdorferi* rapidly replicates but the spirochete load steeply drops during the subsequent molting period [1]. The pathogen again undergoes fast replication in response to a fresh bloodmeal, leading to a surge in the bacterial load in feeding nymphs [2]. Soon after completion of feeding and detachment, the bacterial load decreases and spirochetes clear from organs, such as salivary glands, other than the midgut. The spirochete burden continues to decline when engorged nymphs molt to the adult stage [1]. It is important to control the spirochete population as overgrowth would not only compete for nutrients with the host, but certainly kill it, leading to termination of its life cycle.

Programmed cell death (PCD) broadly refers to any form of cell death mediated by an intracellular death program, including apoptosis, autophagy and programmed necrosis [3], [4]. PCD is crucial in development and homeostasis; all multicellular organisms have a physiologically programmed continuum of pathways to PCD, including animals, plants and fungi [4]–[6]. In recent years PCD has been reported in bacteria, including *Escherichia coli* [7], *Bacillus subtilis* [8], [9], *Staphylococcus aureus* [10], and *Caulobacter cerscentus* [11].

RpoS (*σ* ^38^), one of the two alternative *σ* factors in *B. burgdorferi*, is tightly controlled by multiple regulators, including RpoN and BosR [12]–[14]. Lack of either regulator completely abolishes activation of RpoS. Moreover, RpoS expression is also under influence of small RNAs and Badr [15], [16]. RpoS, in turn, regulates several known important virulence factors, including OspC, DbpA, DbpB, BBA07 and BBK32 [17]–[20], in addition to essential virulence factors that remain to be identified for mammalian infection [21]. In flat ticks, RpoS is not expressed; in response to bloodmeal, *B. burgdorferi* upregulates the *σ* factor, whereby activating RpoS-dependent genes and prepares *B. burgdorferi* for infection of a mammal [22], [23]. During infection of a mammal, *B. burgdorferi* persistently expresses RpoS as it regulates genes that are important for sustaining the infection [24].

Repeated failure to introduce a promoterless *rpoS* gene fused with the *flaB* promoter into a *rpoS* mutant led us to hypothesize that RpoS, when expressed at a high level, is lethal to *B. burgdorferi*. To address this hypothesis, RpoS expression was engineered under the control of an inducible promoter. Inducing RpoS expression caused cell death but no DNA fragmentation was detected during cell death. Cryo-electron microscopy showed induced cells first formed blebs, which were eventually released from dying cells. Further investigation would reveal intracellular programs or/and pathways that govern cell death.

## Materials and Methods

### Construction of pBBE22-*rpoS'*

**Figure 1:**
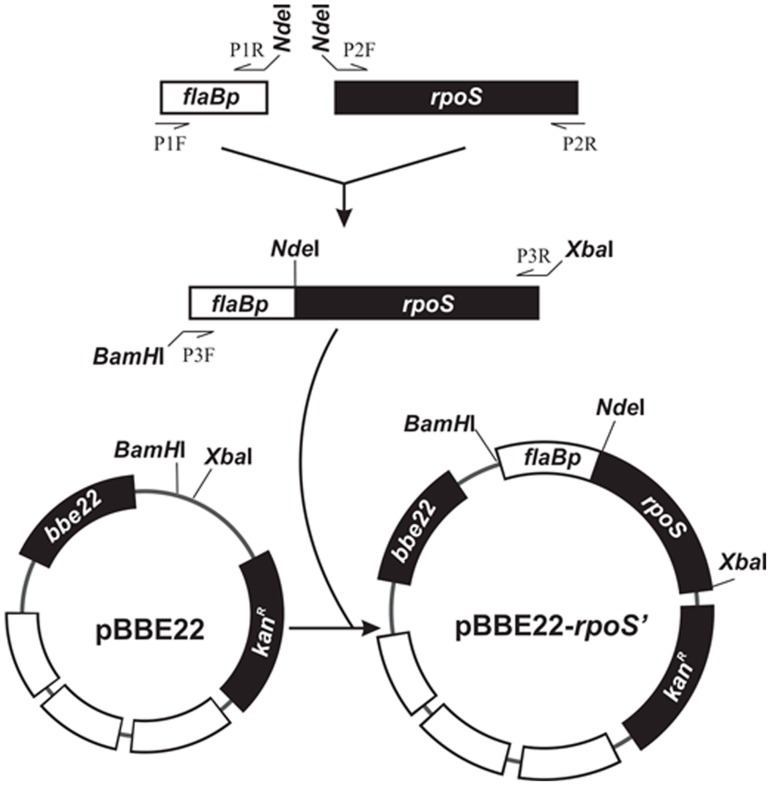
Construction of pBBE22-*rpoS'*. The *flaB* promoter region (*flaBp*) and the promoterless *rpoS* gene were PCR amplified, fused, and cloned into pBBE22.

As illustrated in Fig. 1, a 251-bp fragment of the *flaB* promoter region was amplified with use of a primer pair, P1F and P1R (Table 1). A 944-bp fragment extending from the start codon ATG to the 143-bp sequence downstream of the stop codon of the *rpoS* gene was amplified with use of another primer pair, P2F and P2R. The two PCR products were pooled, purified using the QIAquick PCR Purification Kit (QIAGEN Inc., Valencia, CA), digested with NdeI, repurified, and ligated. The resultant product was used as a template and amplified with the use of a primer pair, P3F and P3R. The amplicon was purified, digested with BamHI and XbaI, and cloned into pBBE22 (a gift from S. Norris) [25]. The insert and flanking regions within the recombinant plasmid were sequenced to ensure the construct was as designed.

**Table 1:** Primers used in the study^a^.

Primer	Sequence (5′ to 3′)^a^
**P1F**	AGAAGTACGAAGATAGAGAGAGAAA
**P1R**	AACACATATGTCATTCCTCCATGATAAA
**P2F**	TACATATGAACATATTTAGTAATGAGG
**P2R**	ACTTGCAAATGCCTGAGTTATTGC
**P3F**	ATAGGATCCAAGATAGAGAGAGAAAAGT
**P3R**	CCTCTAGACCACTGACTTACAAGTAGAC
**P4F**	TAATCTCGAGGCATGCAAGCTTGAAGATAGAGAGAGAA
**P4R**	TATACTCGAGCCATGGTTAATTTCTCCTCTTTAATGAATTC
**P5F**	TTCATGCCATGGACATATTTAGTAATGAGG
**P5R**	TTTCCGCTCGAGACTTACAAGTAGACCAGAACAT

^a^ The underlined sequences are restriction enzyme sites: a BamHI site (P3F), NdeI sites (P1R and P2F), a NcoI site (P5F), and an XbaI site (P3R), and XhoI sites (P4F, P4R and P5R).

### Modification of pJSB104 and construction of pIBM-*rpoS_in_*

**Figure 2:**
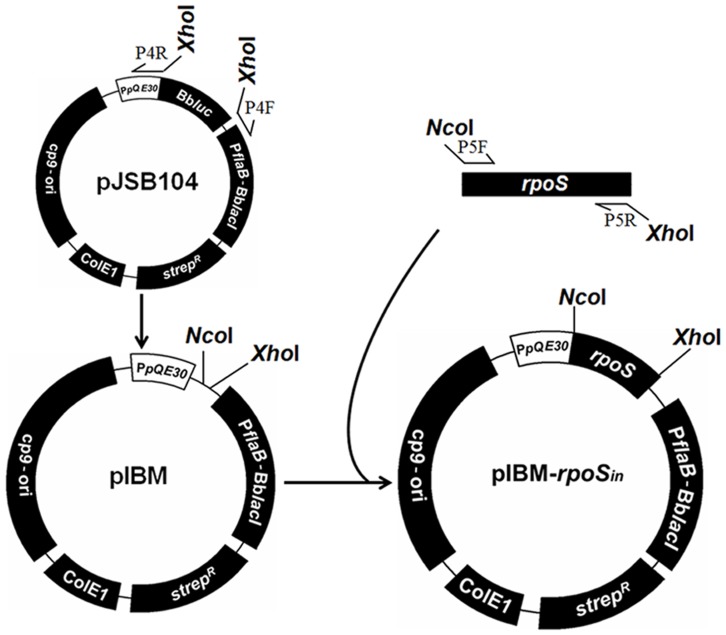
Modification of pJSB104 and construction of pIBM-*rpoS_in_*. pJSB104 was PCR amplified to introduce restriction enzyme sites, digested with XhoI, and circularized to create pIBM. The promoterless *rpoS* gene was PCR amplified and cloned into pIBM.

The construct pJSB104 (a gift from M. Norgard, Texas Southwestern) was first modified to make it easier to accept an insert of interest. pJSB104 was created as described in a previous study [26]. As illustrated in Fig. 2, the construct was amplified with the use of a primer pair, P4F and P4R (Table 1), to introduce two restriction enzyme sites, NcoI and XhoI. The resultant PCR product was digested with XhoI, and circularized to create pIBM. An 839-bp fragment was amplified from the start codon ATG to the downstream 38-bp of the stop codon of the *rpoS* gene with the use of *B. burgdorferi* B31 13A DNA as template and P5F and P5R as primers. The amplicon was purified by using Wizard® SV Gel and PCR Clean-Up System (Promega, Madison, WI), digested with NcoI and XhoI, repurified and ligated to complete construction of pIBM-*rpoS_in_*. The insert and its flanking regions were sequenced to verify the insertion site and sequence.

### Generation of transformants

The *rpoS* mutant, Δ*rpoS*, which was generated in our previous study [21], was grown to late logarithmic (log) phase in Barbour-Stoenner-Kelly H (BSK-H) complete medium (Sigma Chemical Co., St. Louis, MO). Spirochetes were harvested from approximately 40 ml of culture and transformed with pIBM-*rpoS_in_* as described previously [27]. Transformants were identified by PCR using a primer pair specific for the kanamycin cassette and their plasmid content was analyzed as described previously [27].

### Growth rate estimation

The spirochete culture was grown at 33°C to late log phase (approximately 10^8^ cells/ml) in BSK-H complete medium and diluted to 10^4^ cells/ml with the same medium. A total of thirty 1.3-ml aliquots were prepared and isopropyl-beta-D-thiogalactopyranoside (IPTG) was then added final concentrations at 0, 0.004, 0.008, 0.016, 0.032, 0.063, 0.125, 0.25, 0.5 and 1.0 mM. Each concentration was in triplicate. All aliquots were incubated at 33°C and cell numbers were counted daily for 20 days.

### Immunoblotting

The spirochete culture was grown at 33°C to late log phase (approximately 10^8^ cells/ml) in BSK-H complete medium, diluted to 10^4^ cells/ml and divided to six 5.0-ml aliquots before IPTG was added to final concentrations at 0, 0.004, 0.008, 0.016, 0.032 and 0.063 mM. All aliquots were cultured for 8 days and cells were harvested by centrifugation at 5,000×*g* at 4°C. Resultant pellets were dissolved in a SDS-PAGE sample buffer, separated by electrophoresis and electrotransferred onto nitrocellulose membrane. Blots were probed either with a mixture of FlaB, OspA and OspC mAbs, or anti-RpoS MAb alone as described in our previous study [28]. Anti-RpoS MAb was kindly provided by F. Yang (Indiana University School of Medicine).

### DNA extraction and electrophoretic analysis

Spirochetes were grown to early log phase (approximately 10^7^ cells/ml) in BSK-H medium at 33°C before IPTG was added to a final concentration at 1.0 mM. After induction for 2 days, bacteria were harvested by centrifugation. Untreated cells were collected as a control. Total DNA was prepared as described previously [29], and quantified using ND-1000 Spectrophotometer (NanoDrop Technologies, Inc., Wilmngton, DE). A total of 120 ng DNA was loaded to a 0.4% agarose gel. Two sets of DNA ladders, λ DNA-HindIII digest (NEB Co., Ipswich, MA) and 1 kb Plus DNA ladder (Invitrogen Co., Grand Island, NY), were used as markers. The former ladder contained DNA with mass of 23, 9.4, 6.5, 4.4, 2.3 and 2.0 kbp while the later consisted of DNA of 12, 11, 10, 9, 8, 7, 6, 5, 4, 3, 2, 1.65, 1, 0.85, 0.65, 0.5, 0.4, 0.3, 0.2, 0.1 kbp. Electrophoresis was performed either under 80 volts for 2 h for examination of DNA fragmentation or 25 volts for 19 h for investigation of the integrity of genomic DNA. Gels were then stained with GelRed™ (Biotium Co., Hayward, CA) for 30 min and imaged under UV light.

### Terminal deoxynucleotidyltransferase dUTP nick end labeling assay

Spirochetes were grown at 33°C to early log phase (approximately 10^7^ cells/ml) in 12 ml of BSK-H medium. The culture was divided into two; IPTG was added to one aliquot to a final concentration of 1.0 mM and the second was used as the control. After 2 days of induction, cells were harvested by centrifugation at 3,000×g for 10 min at 4°C and washed twice with PBS. Resulting cell pellets were subjected to DNA fragmentation analysis with the use of the APO-DIRECT™ Kit (BD Biosciences, San Jose, CA) per the manufacturer's instructions. Labeled cells were examined and imaged with 535 and 480 nm in wavelength, respectively, under Olympus IX71 Microscope (Olympus America Inc., Melville, NY).

### Cryo-electron microscopy

Spirochetes were grown to early log phase (approximately 10^7^ cells/ml) in BSK-H medium at 33°C before IPTG was added to a final concentration at 1.0 mM and allowed to induce for 3 days. Bacteria were harvested from 1.0 ml of culture treated either with or without the inducer by centrifugation at 5,000×*g* for 5 min, suspended in 20 µl PBS and then deposited onto freshly glow-discharged holey carbon grids for 1 min. Grids were blotted with filter paper and then rapidly frozen in liquid ethane, using a homemade gravity-driven plunger apparatus. The frozen-hydrated specimens were imaged at −170°C using Polara G2 electron microscope equipped with a field emission gun and a 16 megapixel CCD camera (FEI Co., Hillsboro, OR). The microscope was operated at 300 kV. The low mag images were collected at high defocus to achieve high contrast.

### Statistical analysis

*P* values were calculated using GraphPad Prism 5.0 (GraphPad Software Inc., La Jolla, CA).

## Results

### Transformation of an *rpoS* mutant with an *rpoS* gene fused with the *flaB* promoter was unsuccessful

To generate a variant with constitutive RpoS expression, pBBE22-*rpoS*' was constructed by cloning a promoterless *rpoS* gene fused with the *flaB* promoter into pBBE22, in which *rpoS* expression was designed under control of the *flaB* promoter (Fig. 1). Repeated attempts to introduce pBBE22-*rpoS*' to Δ*rpoS* were unsuccessful, leading us to hypothesize that a high level of RpoS expression is lethal to *B. burgdorferi*.

### Induction of spirochete death results from increasing RpoS expression

To address the hypothesis that RpoS is lethal when expressed at a high level, pIBM-*rpoS_in_* was constructed as illustrated in Fig. 2. Within the construct, RpoS expression was under the control of an inducible promoter; thus no RpoS production should occur in the absence of the inducer IPTG. As expected, pIBM-*rpoS_in_*, unlike pBBE22-*rpoS*', was easily introduced into Δ*rpoS*. In a single transformation experiment, five transformants were obtained. Plasmid analyses led to identification of one clone, namely, Δ*rpoS/rpoS_in_*, which lost cp9, lp5, lp21, lp25 and lp56 as Δ*rpoS*. Although Δ*rpoS* lost several plasmids, the mutant was restored with full infectivity after complementation [21].

**Figure 3:**
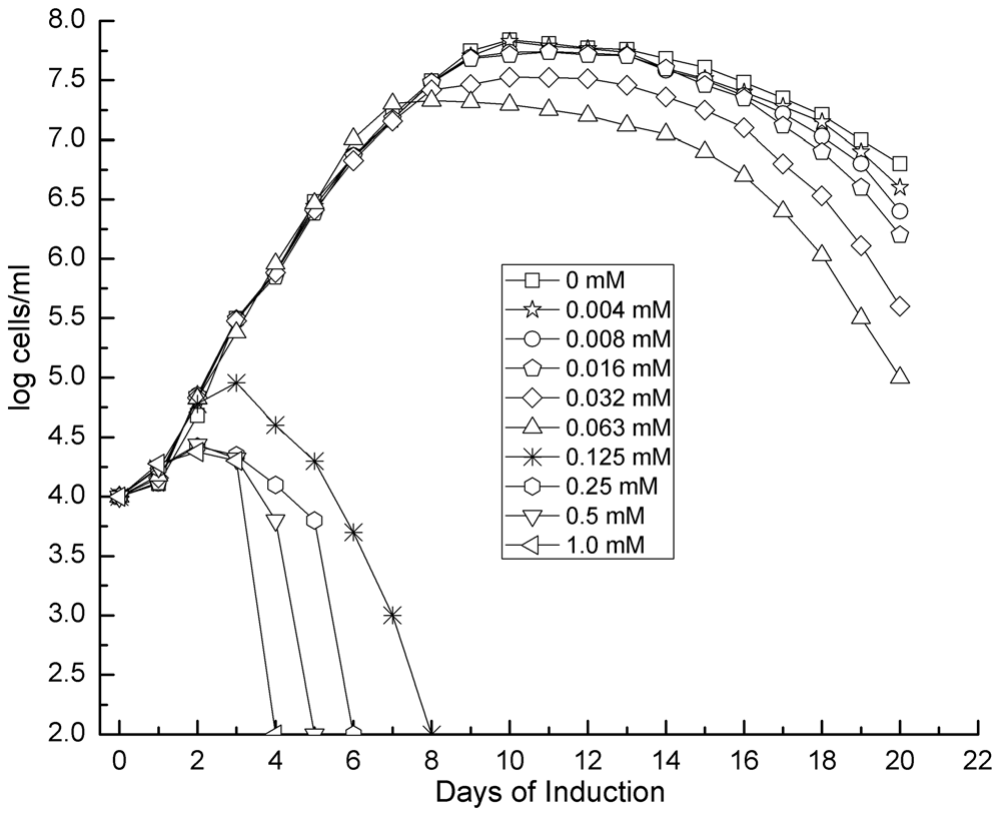
Induction of spirochete death by inducing RpoS expression. A total of thirty 1.3-ml aliquots of Δ*rpoS/rpoS_in_* spirochetes at a density of 10^4^ cells/ml were prepared and supplemented with IPTG to final concentrations at 0, 0.004, 0.008, 0.016, 0.032, 0.063, 0.125, 0.25, 0.5 and 1.0 mM. Each concentration was in triplicate. The 30 aliquots were incubated at 33°C and cell numbers were counted daily for 20 days. Mean counts presented here are calculated from the triplicates of each treatment.

The transformant Δ*rpoS/rpoS_in_* was grown to early log phase (10^7^ cells/ml) in BSK-H medium and diluted to 10^4^ cells/ml before IPTG was added to final concentrations ranging from 0.004 to 1.0 mM in a 2-fold increment. When the IPTG concentration was at 0.125 mM or higher, not was spirochete growth inhibited, but cells were killed within 4 to 8 days (Fig. 3). This inhibiting and killing effect was enhanced as the inducer concentration increased from 0.125 to 1.0 mM. Although at lower concentrations IPTG did not affect early growth, its presence reduced the stationary cell density.

A paucity of evidence exists regarding the toxicity of IPTG directly related to *B. burgdorferi* [26], [30]. Nevertheless, to rule out any possibility that the inducer can kill spirochetes in the absence of the inducible promoter, IPTG was also added to both Δ*rpoS* and Δ*rpoS/rpoS* cultures. At a 1.0 mM concentration, the inducer did not interfere with growth at all (data not shown).

**Figure 4:**
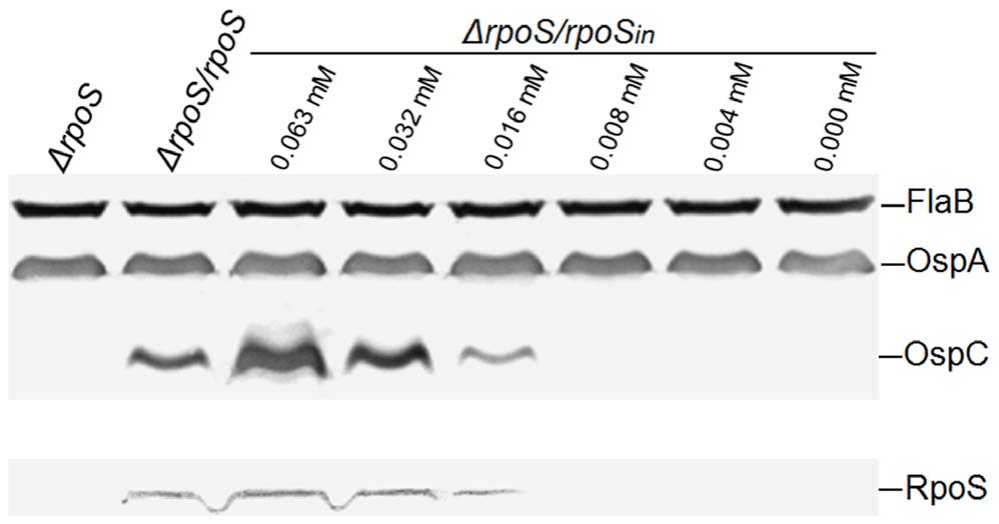
IPTG induces RpoS and OspC expression. A total of six 5.0-ml aliquots of Δ*rpoS/rpoS_in_* spirochetes at a density of 10^4^ cells/ml were prepared, supplemented with IPTG to final concentrations at of 0, 0.004, 0.008, 0.016, 0.032 and 0.063 mM, and induced for 8 days. Δ*rpoS* and Δ*rpoS/rpoS* cells were used as controls. Bacteria were harvested and analyzed using immunoblotting probed with a mixture of anti-FlaB, OspA and OspC antibodies (upper panel), and RpoS antibody alone (lower panel), respectively.

The killing activity induced by IPTG was most likely realized through increasing RpoS expression. To correlate RpoS expression with an increase in inducer concentration, RpoS expression was further examined. A concentration of 0.008 mM or lower, IPTG was not able to induce RpoS expression to a detectable level (Fig. 4, lower panel); however, at a 0.016 mM concentration, the inducer induced a significant amount of expression, but lower than the activity driven by its native promoter. At concentrations of 0.032 mM or higher, IPTG induced a stronger RpoS expression than the native promoter. When the concentration reached 0.125 mM or higher, bacteria were killed; consequently no antigens were harvested for immunoblot analysis. The data presented in Fig. 4 also demonstrated that RpoS expression activity determined that of OspC, not OspA.

### Induced cells undergo quicker division before death

**Figure 5:**
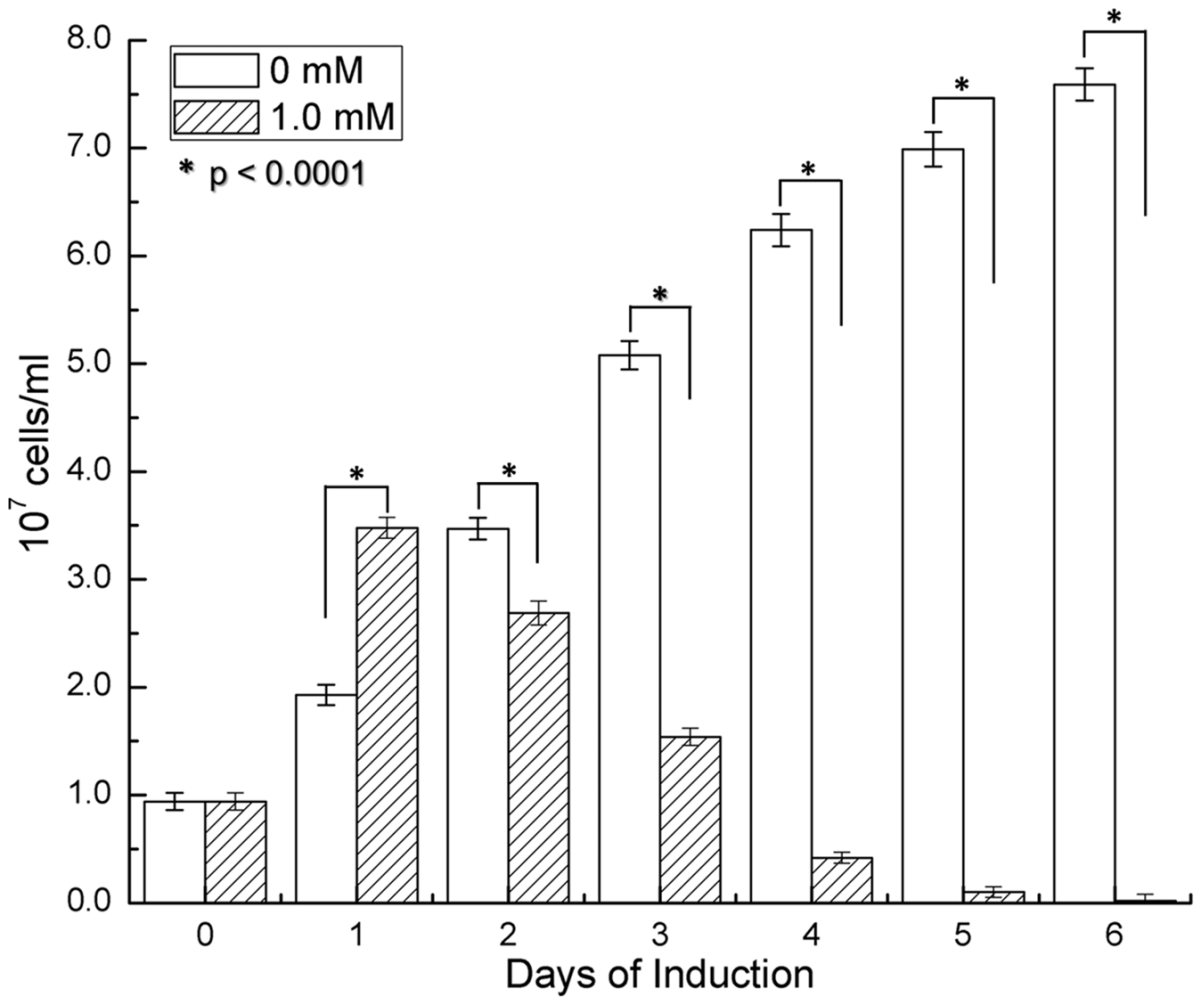
IPTG stimulates replication before causing death. Six 1.3-ml aliquots of Δ*rpoS/rpoS_in_* spirochetes at a density of 10^7^ cells/ml were prepared. IPTG was added to 3 aliquots for a final concentration of 1.0 mM. All aliquots were incubated at 33°C and bacterial numbers were counted daily for 6 days. Means and standard deviations presented here are calculated from triplicates.

While generating data presented in Fig. 3, spirochete numbers underwent a noticeable upsurge immediately after the addition of IPTG. However, due to the log scale and initially low bacterial numbers after induction, these data had not been shown clearly. To overcome the issue, Δ*rpoS/rpoS_in_* cultures grown to 10^7^ cells/ml were supplemented either with 1.0 mM IPTG or nothing as a control. After 24 h, the number of treated cells was 80% higher than the untreated (*P*<0.0001) (Fig. 5), indicating that the induced cells had undergone quicker division than the untreated ones. On day 2, the trend reversed as the treated cells began dying, resulting in a 22% decrease in total number when compared to the untreated cells, which continued to replicate (*P*<0.0001). Steadily declining in number, by day 6, the treated cells essentially became uncountable, supporting the data reported in Fig. 3, and further demonstrating that the IPTG treatment causes cell death.

### No DNA fragmentation occurs during induction of cell death

**Figure 6:**
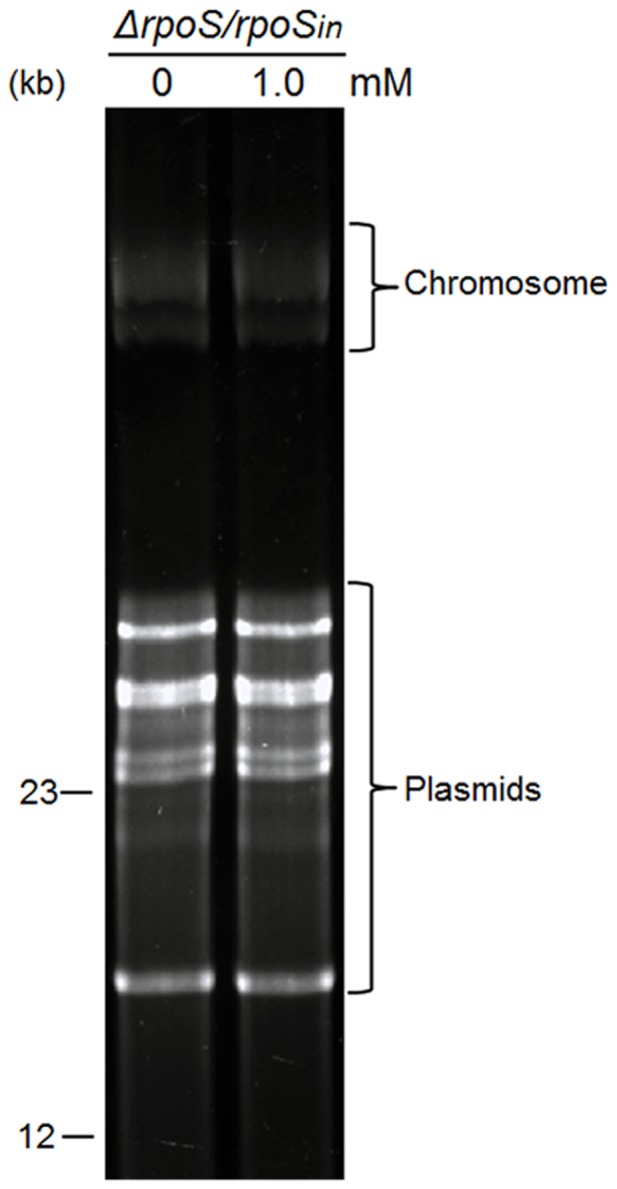
Genomic DNA remains intact in induced cell death. The transformant Δ*rpoS/rpoS_in_* was grown to early log phase (10^7^ cells/ml) before IPTG was added to a final concentration of 1.0 mM and induced for 2 days. Spirochetes not receiving IPTG were used as the control. Total DNA was extracted and analyzed by electrophoresis. Two DNA markers (12 and 23 kb) are labeled on the left.

One hallmark of PCD is DNA fragmentation. To investigate whether induced cell death results from DNA degradation, Δ*rpoS/rpoS_in_* spirochetes were induced with IPTG, then total DNA was extracted and analyzed. Electrophoresis for 2 h showed no DNA fragmentation occurring (data not shown); therefore, electrophoresis was increased to 19 h in order to separate large genomic DNA. As shown in Fig. 6, no difference in DNA pattern was detected between induced and control cells, indicating that the genomic DNA, including both chromosome and plasmids, remained intact.

**Figure 7:**
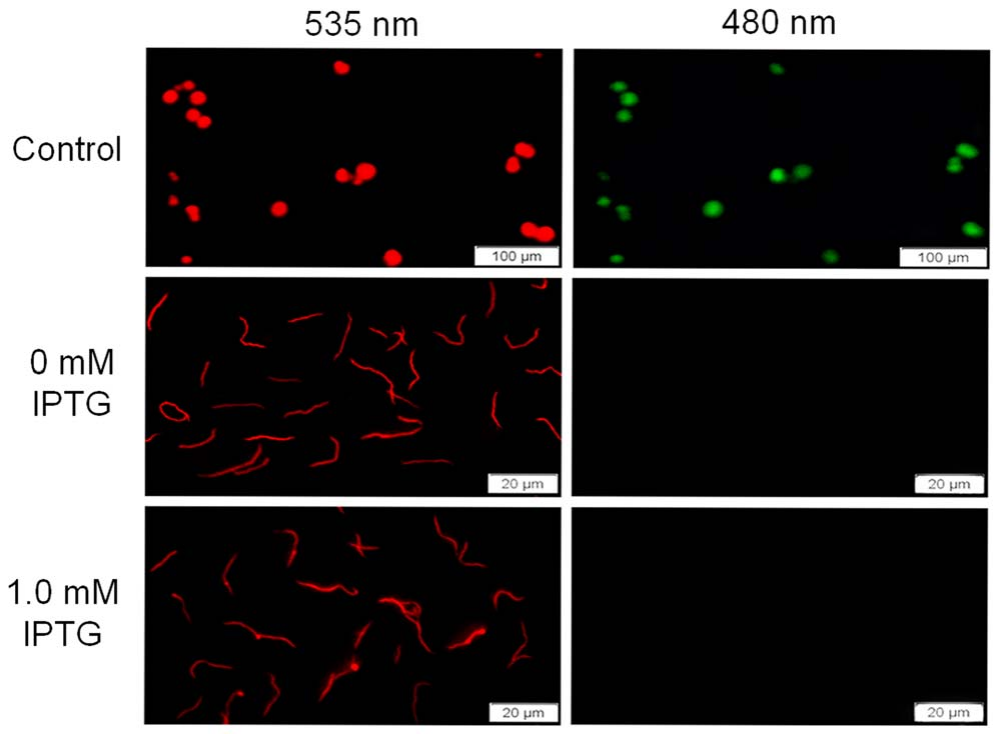
No DNA breaks are detected in induced cell death. IPTG-induced and uninduced spirochetes were labeled with the terminal deoxynucleotidyltransferase dUTP nick end labeling assay. Labeled cells were examined and imaged under the Olympus IX71 Microscope with 535 and 480 nm wavelengths, respectively. The positive control cells provided in the labeling kit were presented in the top panel.

The terminal deoxynucleotidyltransferase dUTP nick end labeling assay, a single step staining method for tagging DNA breaks, was used to further rule out the possibility of DNA fragmentation through the detection of apoptotic cells. IPTG-induced Δ*rpoS/rpoS_in_* cells showed negative imaging as untreated spirochetes under wavelength of 480 nm (Fig. 7). The inducer did not increase the number of 3′-termini of genomic DNA, again demonstrating that induced cell death does not undergo DNA fragmentation.

### Dying cells form blebs

**Figure 8:**
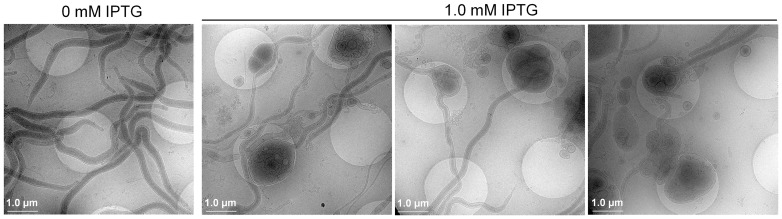
Spirochetes form blebs before death. Δ*rpoS/rpoS_in_* spirochetes were induced with IPTG at a final concentration of either 0 or 1.0 mM for 3 days and imaged using the Polara G2 electron microscope.

To explore how induction causes cell death, induced cells were examined using cryo-electron microscopy. IPTG induced an intensive formation of blebs, which formed further small bodies in various sizes; these were eventually released from parental spirochetes (Fig. 8). Apparently blebbing disintegrated cells and eventually caused death.

## Discussion

The current study showed that increasing RpoS expression facilitates cell division before causing death. Although investigations into RpoS regulation and RpoS-dependent genes have been intensively conducted since Norgard and colleagues initially showed that the alternative σ factor regulates important virulence determinants more than a decade ago [13], it is far from understood how increasing its expression can promote cell division and induce cell death. Under normal conditions, *B. burgdorferi* does not express RpoS during rapid growth; conversely it starts to accumulate RpoS as cells enter later log phases [13]. Initial microarray analysis suggested RpoS may regulate over a hundred genes [31]–[34]. Thus far, however, very few genes have been confirmed to be RpoS-dependent with most encoding for outer surface lipoproteins and associated mammalian infection [13], [17], [35]–[37]. Increasing expression of these genes should certainly not cause cell death [28], [38], [39]. The RpoS-regulated genes are much better understood in *E*. *coli*, albeit more investigation is still needed. For example, RpoS is involved in the regulation of the *ftsQAZ* operon, whose products play a role in the timing of cell division [40]. The homologue of FtsQAZ, including FtsA, FtsH, FtsW and FtsZ, can be found in *B. burgdorferi* [41]. Further investigation is needed to elucidate the RpoS-regulated genes in *B. burgdorferi* and whether quick cell division by inducing RpoS is achieved through influencing expression of FtsA, FtsH, FtsW and/or FtsZ.

Cryo-electron microscopy showed that induced cells formed blebs before death. During apoptosis in eukaryotic cells, blebs form as the cell's cytoskeleton breaks up and causes the membrane to bulge outward [42]. These bulges may separate from the cell by taking a portion of cytoplasm to produce apoptotic bodies. *B. burgdorferi* does not have a cytoskeleton; thus its blebbing must follow a different path. It is unknown if promoting spirochete division alone can cause blebbing. In *E. coli*, RpoS-dependent genes, involved with changes in cell membrane permeability and general cell morphology, mostly belong to the *osm* family of genes [43]. These membrane proteins are tightly regulated as they are important to the maintenance of cell permeability and morphology. Likewise, several outer membrane proteins, including P66, BmpA, BmpB and BmpC, are identified in *B. burgdorferi* [44]–[49]. However, it remains to be seen whether increasing RpoS expression results in an imbalanced expression of these membrane proteins, consequently inducing abnormal spirochetal morphology causing blebbing and eventually cell death.

After acquisition by the tick vector and following bloodmeal, *B. burgdorferi* rapidly replicates, a process that may efficiently help store nutrients. In the event of a 10-fold drop in spirochete abundance during the subsequent molting period [1], [2], *B. burgdorferi* may disintegrate via blebbing, a process that releases nutrients for use in the host and bacteria. Regardless of the mechanism by which *B. burgdorferi* proceeds to cell death, bacterial demise by means of blebbing may provide easily as sample nutrients for both spirochetes and host as blebbing produces smaller components, theoretically easier to be digested and absorbed in comparison with the whole spirochete body. These are also issues that remain to be investigated.

The current study showed that *B. burgdorferi* undergoes rapid division, leading to a surge in bacterial numbers within the first day post-induction. Although induced cells started dying on day 2, the total cell count remained higher on day 3 than pre-induction. This observation may explain why induced cell death was not noted in a pioneering study by Samuels and colleagues, who successfully used an inducible promoter for the regulation of RpoS expression, in which all data were obtained within 72 h post-induction with ITPG [30]. The current study showed that the total cell number did not show a decline until day 3 post-induction although induced cells started dying within 48 h.

PCD facilitates the removal of aged, damaged, infected, problematic or unnecessary cells, and is a vital process in the development and homeostasis of eukaryotic multicellular organisms, including animals, plants and fungi [4]–[6]. In bacteria, PCD is counterproductive for an individual cell; however, it might be advantageous for a whole population [3]. For example, *mazEF*-mediated death can act as a defense mechanism that prevents the spread of phages in *E. coli* [7]. Bacterial cell death mediated by *mazEF* may have several other roles; the *mazEF* system might act as a guardian of the bacterial chromosome [50], [51]. When, for example, DNA repair systems fail to overcome excess damage of the chromosome, *mazEF*-mediated cell death might be activated. Thus, by eliminating cells that carry genomic defects and mutations, the *mazEF* system might contribute to the maintenance of genomic stability of the whole population. Cell death mediated by *mazEF* may also be important in the response of bacteria to severe nutritional stress [52]. The second PCD program is found in *B*. *subtilis*, in which the *skf* and *sdp* operons mediate the death of a subpopulation of sporulating cells [8], [9]. PCD is also important for biofilm development in *S. aureus* [10]. Following cell death, a sub-population of the dead bacteria lyse and release genomic DNA, which then has an essential role in intercellular adhesion and biofilm stability.

*B. burgdorferi* maintains its life cycle by traveling between the tick vector and a mammal. It may need an intracellular death program more than other microorganisms, especially during the life cycle in the tick vector. Without control of growth, spirochetes would not only compete for nutrients with the host but would certainly kill it. The findings lead us to hypothesize that increasing RpoS expression triggers one or multiple intracellular programs or pathways that eventually cause cell death. Further studies should aid in revealing these programs or pathways that govern cell death in the Lyme disease agent.
